# Oxcarbazepine-Induced Hyponatremia: A Case Report and Comprehensive Literature Review

**DOI:** 10.7759/cureus.15085

**Published:** 2021-05-18

**Authors:** Chidinma Ejikeme, Sherif Elkattawy, Fisayo Kayode-Ajala, Suha Abuaita, Maria Khazai

**Affiliations:** 1 Internal Medicine, Rutgers New Jersey Medical School/Trinitas Regional Medical Center, Elizabeth, USA; 2 Internal Medicine, Trinitas Regional Medical Center, Elizabeth, USA; 3 Internal Medicine, St George's University, True Blue, GRD; 4 Nephrology, Trinitas Regional Medical Center, Elizabeth, USA

**Keywords:** siadh, hyponatremia, oxcarbamazepine, electrolyte disturbances, anti-convulsant

## Abstract

Oxcarbazepine is a well-known and effective anti-convulsant used for patients with underlying seizure disorder. It is a structural analog of carbamazepine; however, it follows a different metabolic pathway in which it is converted to a different active metabolite. Side effects associated with this medication are vast; however, in this report, we will hone in on the renal adverse effects, e.g., syndrome of inappropriate anti-diuretic hormone secretion (SiADH). SiADH is a condition in which the body is making too much anti-diuretic hormone, which, in turn, results in "too much" water absorption, causing hyponatremia with neurologic sequelae. Our patient is a 31-year-old gentleman with a history of depression, anxiety, bipolar disorder, and previous suicide attempts who presented to the emergency department following oxcarbazepine overdose and was subsequently found to be hyponatremic secondary to having SiADH.

## Introduction

Oxcarbazepine is an anti-convulsant that has been used for years as a treatment for seizures and trigeminal neuralgia. It was discovered in 1953 but marketed in 1963 to treat epilepsy [1]. As a drug, it works on binding voltage-gated sodium channels in their inactive form, thereby preventing sustained or repetitive firing of an action potential [1,2]. Due to its mechanism of action, oxcarbazepine toxicity is associated with neurologic, cardiovascular, and anticholinergic symptoms [3]. This report highlights a case of oxcarbazepine-induced SiADH in a 31-year-old male with a medical history significant for depression, anxiety, and bipolar disorder. This case explores the side effects of carbamazepine toxicity with emphasis on hyponatremia secondary to SiADH.

## Case presentation

A 31-year-old male with a past medical history of depression, anxiety, bipolar disorder, and previous suicide attempts presented to the emergency department (ED) following an unknown drug overdose. The patient was confused on initial presentation; hence, he could not provide further history. The patient's family explained that the patient had complained of nausea, subjective fever, and chills two days ago. He was scheduled for a SARS-CoV-2 (severe acute respiratory syndrome coronavirus 2) screening test on the day of presentation. Before his appointment, he had “multiple” cans of beers, and then lost his way and called his family for help. While on the phone with family, the patient stated that "he did not wish to live anymore." Upon arriving home, the patient took about 75 pills of his oxcarbazepine, which was prescribed for his underling psychiatric disorders as mentioned above. The patient was later found unresponsive by his family and brought to the ED for further evaluation.

**Figure 1:**
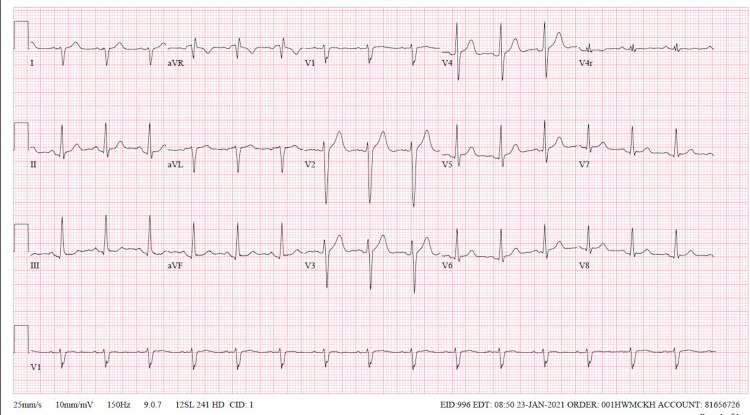
EKG showed sinus tachycardia with right bundle branch block EKG, electrocardiogram

**Figure 2:**
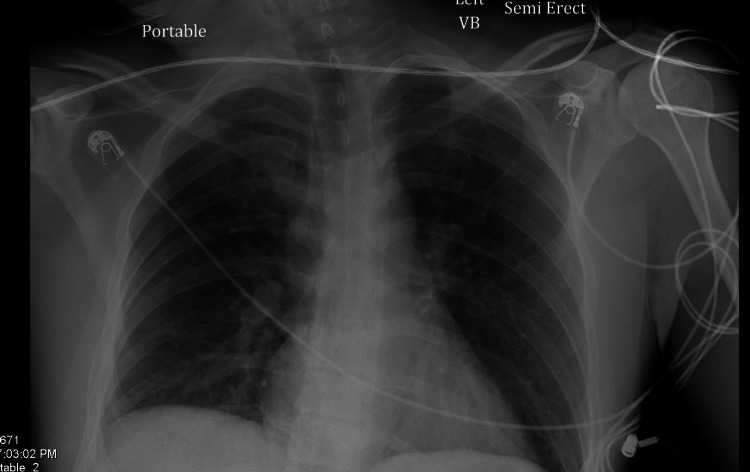
Portable chest X-ray significant for patchy increased density in the right base

Upon arrival to the ED, initial vital signs revealed a temperature of 98.2°F, heart rate of 76 beats/minute, respiratory rate of 16 breaths/minute, blood pressure of 164/78 mmHg, and oxygen saturation of 100%. Physical examination was significant for mild bibasilar crackles. The patient was somnolent; however, he was able to follow commands with a Glasgow coma scale of 12. There were no neurological deficits. The rest of his physical examination was normal. A standard urine drug screen was performed, which was negative. Lab findings revealed an alcohol level of 295.5 mg/dL with a creatine phosphokinase (CPK) level of 186 U/L. Electrocardiogram (EKG) showed sinus tachycardia with right bundle branch block, as seen in Figure 1. It was difficult to comment on QTC interval given tachycardia. Basic metabolic panel was significant for a sodium level of 133 mmol/L (reference range: 135-145 mmol/L). A serum oxcarbazepine level was performed, which was elevated at 65.6 mcg/mL. Chest X-ray showed patchy increased density in the right base, as seen in Figure 2.

**Figure 3:**
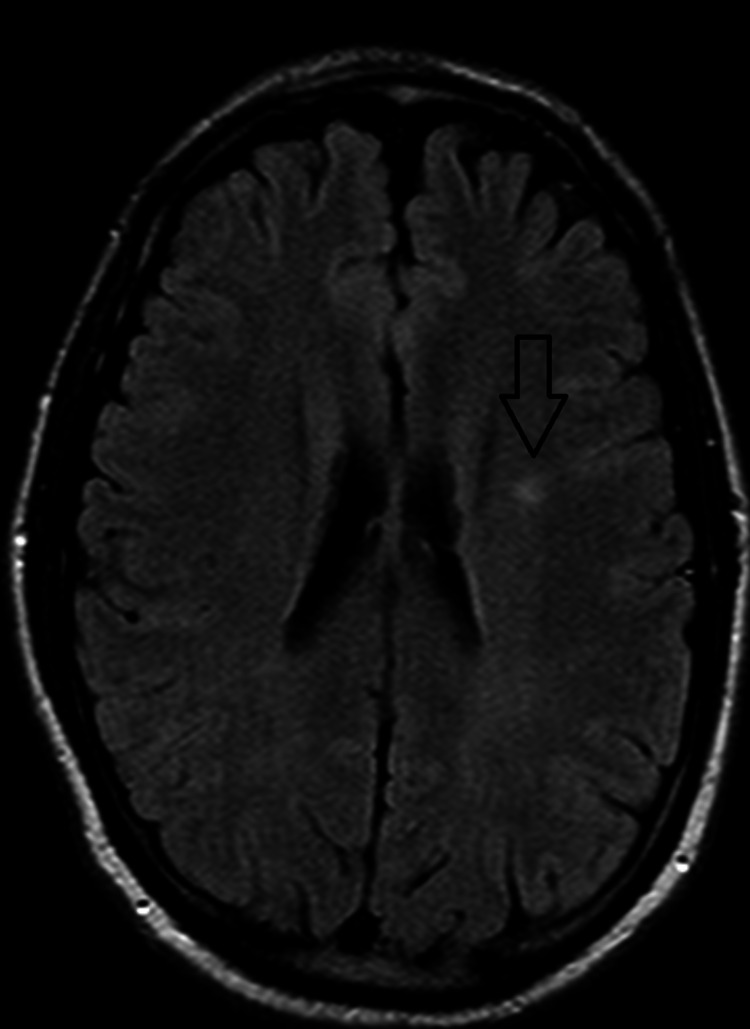
MRI of the head shows a small nonspecific focus of increased signal intensity in the left posterior frontal periventricular white matter, possibly representing a small focus of demyelination, chronic ischemia, or gliosis

The patient had an episode of generalized tonic-clonic seizure, which improved after receiving 1 mg IV push of lorazepam. The patient was not intubated as he had a Glasgow coma scale of 10. He was continued on isotonic fluid and was transferred to the intensive care unit (ICU) for closer monitoring. While in the ICU, poison control was contacted and recommended continuing supportive care and performing periodic EKGs to monitor for QRS and QTC interval changes. CIWA (Clinical Institute Withdrawal Assessment of Alcohol Scale) protocol was initiated and the patient received thiamine and folic acid. The patient was also placed on seizure and fall precautions and was started on levetiracetam by the neurology team. Electroencephalogram (EEG) was performed, which showed occasional epileptiform discharges. MRI of the head showed a small nonspecific focus of increased signal intensity in the left posterior frontal periventricular white matter, possibly representing a small focus of demyelination, chronic ischemia, or gliosis, as seen in Figure 3. Additionally, the patient was placed on one-to-one observation, and the psychiatry team was consulted for a suicide attempt.

The patient's mentation improved with supportive treatment. Subsequent EKGs performed did not show QTC prolongation; however, the patient's sodium level decreased to 129 mmol/L. Urine/serum osmolality and urine electrolytes were performed to evaluate for his worsening hyponatremia. Serum osmolality was 264 mosm/kg, with a urine sodium level of 110 mmol/L, urine chloride of 97 mmol/L, and urine osmolality 601 of mosm/kg, which suggests syndrome of inappropriate anti-diuretic hormone (SiADH). Urinalysis showed a specific gravity of 1.020.

The patient was started on fluid restriction, and his sodium level was monitored every six hours. Sodium level decreased to as low as 123 mmol/L and then gradually titrated upward with fluid restriction. The patient remained awake, alert, and oriented, and asymptomatic during his hospital stay. The last basic metabolic panel showed a sodium level of 138 mmol/L. He was cleared by the primary team for discharge and transferred to the psychiatry unit involuntarily for further evaluation.

## Discussion

Oxcarbazepine is an anti-convulsant drug that is administered orally as an extended-release tablet or as an oral suspension in order to treat seizures, nerve pain, and bipolar disorder. Oxcarbazepine stimulates the collecting tubule V2 receptor-G protein complex independent of anti-diuretic hormone (ADH), leading to increased renal tubular water absorption [1]. These channels are found on the collecting ducts and they carry water across the membrane into the bloodstream [2]. Oxcarbazepine has been linked to Steven Johnson syndrome, pancreatitis, aplastic anemia, angioedema, impaired concentration, asthenia, and ataxia. In this report, we will focus on the renal complication, SiADH [3]. SiADH, as the name implies, is an entity that involved excessive production of ADH, also known as vasopressin. This hormone allows the body to retain water. One of the most feared yet common complication of SiADH is hyponatremia. Hyponatremia can present with symptoms such as nausea, vomiting, headaches, confusion, fatigue, restlessness, and muscle weakness. If severe enough it may also lead to seizures and possibly coma [2].

Oxcarbazepine-induced SiADH is a result of an increase in ADH, which increases sensitivity of the aquaporin 2 channels in the renal tubules, resulting in hyponatremia. Regarding management, fluid restriction has been proven to correct hyponatremia. If severe enough, hypertonic saline and demeclocycline are also correlated with an improvement in symptoms [4]. In our case, fluid restriction was sufficient to bring the sodium levels back to normal.

One of the most common side effects of oxcarbazepine is nausea and vomiting, which according to some studies is secondary to hyponatremia [5,6]. Hyponatremia caused by oxcarbazepine has been linked to patients with epilepsy, neuralgia, and psychological disorders. Our patient had bipolar disorder and presented with hyponatremic symptoms including nausea, vomiting, and headaches. In severe forms, hyponatremia presents with altered mental status and demyelination syndrome. Gastrointestinal and neurologic symptoms have been found in multiple studies in patients with oxcarbazepine-induced SiADH [5]. Other side effects of oxcarbazepine include seizures, respiratory distress, lethargy, headache, and altered mental status [6].

SiADH is caused by one of the following four major categories: pulmonary disorders, malignancy, neurologic disorders, and medications. Other medications commonly associated with SiADH include chlorpropamide, carbamazepine, and cyclophosphamide. Studies have shown that chlorpropamide increases sodium permeability in the loop of Henle, causing more water absorption in the collecting ducts by increasing the number of ADH receptors, whereas oxcarbazepine, which is structurally related to carbamazepine, increases the sensitivity of the ADH receptors [4-6]. Even though our patient had a patchy increased density in the right lower lobe on chest X-ray, he was not treated with any antibiotics during his stay given no objective fevers or symptoms during his hospitalization.

SiADH also holds a diagnostic criteria, which includes a urine osmolality of more than 100 mOsm/kg and urinary sodium of more than 40 mmol/L. Several cases have also associated these diagnostic findings in relation to SiADH [6]. Our case fulfilled the aforementioned diagnostic criteria with serum osmolality of 264 mOsm/kg and urine sodium of 110 mmol/L. The clinical manifestations have been correlated with the severity of hyponatremia [7]. Nausea and vomiting have been shown to correlate with sodium levels below 125 to 130 mEq/L. Coma and respiratory distress have been correlated with serum sodium levels below 115 to 120 mEq/L. Other symptoms include gait disturbances, memory and cognitive disturbances, fatigue, dizziness, confusion, and muscle cramps [7]. In our particular case, our patient presented with nausea, vomiting, fever, and chills, and later on developed seizure-like episodes. Studies have previously shown that the discontinuation of the culprit medication and fluid restriction have gradually increased sodium levels. In our particular case, the sodium levels normalized gradually with fluid restriction.

## Conclusions

Oxcarbazepine-induced SiADH is a well-known entity that can present with morbid outcomes in the setting of severe hyponatremia. The causes of SiADH are broad, and clinicians should tailor the treatment of hyponatremia to the cause of SiADH for better patient outcomes. Furthermore, hyponatremia as a result of SiADH is common; however, attempts should be made to find the underlying cause instead of pursuing fluid restriction to reduce patient re-hospitalization and hospital length of stay.
